# Relationship between Peer Pressure and Risk of Eating Disorders among Adolescents in Jordan

**DOI:** 10.1155/2018/7309878

**Published:** 2018-09-19

**Authors:** Nihaya A. Al-sheyab, Tamer Gharaibeh, Khalid Kheirallah

**Affiliations:** ^1^Faculty of Nursing, Maternal and Child Health Department, Jordan University of Science and Technology, PO BOX 3030, Irbid 22110, Jordan; ^2^Faculty of Nursing, Jordan University of Science and Technology, PO BOX 3030, Irbid 22110, Jordan

## Abstract

**Background:**

The prevalence of disordered eating behaviors (DEBs) have increased worldwide. It is estimated that about 31.6% of Jordanian adolescents developed DEB. Engaging in peer groups is a prominent event in which adolescents try to belong to peers as part of exploring their social identity.

**Purpose:**

To assess the relationship between risk of eating disorders and peer pressure among adolescents.

**Methods:**

A descriptive, correlational, cross-sectional design utilized multistage cluster sampling technique was used to recruit students from 8th to 10th grades from both sexes from schools in northern Jordan. Data were collected from a self-administered, online questionnaire which was given to 738 participants.

**Results:**

The difference in overall mean of the Inventory of Peer Influence on Eating Concerns (I-PIEC) between adolescents with disordered eating behaviors and normal eating behaviors states was statistically significant. Scores for interaction peer pressure means were statistically higher for girls than for boys; conversely, likeability mean scores were statistically higher for boys than girls.

**Conclusions:**

The current findings suggest that healthcare professionals are encouraged to conduct appropriate school-based primary prevention for disordered eating behaviors.

## 1. Introduction

Disordered eating behaviors (DEBs) are defined as unhealthy eating action and habit to maintain body weight that involve strict dietary habit [1] and failure to maintain appropriate eating behavior [2]. Recently, DEB has become an issue worldwide, especially among adolescents [3]. The DEBs are relatively high among adolescents. It is estimated about 54.9% among Norwegian adolescents [4], 26.4% in Turkey [5], 14.4% in USA, 23.4% in Emirates [6], and 31.6% in Jordan [7, 8] have engaged in at least one DEB including dieting, binge eating, fasting, skipping meals, or excessive exercising.

During adolescence, DEB tends to increase as a result of changes in psychological, biological, and environmental-social aspects [9, 10]. Biological factors include body mass index (BMI) and pubertal timing [11]. For example, it was found that pubertal phase was an important factor to develop disordered eating among large community male and female samples aged 13 to 16 years [12]. This result was in line with a longitudinal study that had been conducted over 8 years on 1430 adolescents [11]; the association between pubertal development and disordered eating behaviors was mainly assessed at three points, when participants aged from 13 to 14 years, 16 to 17 years, and 19 to 20 years; the result showed that pubertal development was a predictor for disordered eating behaviors during early and middle adolescence. Furthermore, advanced pubertal development predicted dieting during late adolescence. Psychological factors involve positive or negative impact on perception of self-esteem [13]. A cross-sectional study had been conducted on females revealed that self-esteem is a predictor factor for disordered eating behaviors but not considered primary predictor; however, it seems to play an important influential role on the type of eating disorders. For example, it was found that low self-esteem with stress is a primary predictor for bulimia nervosa [14].

Extrinsic factors depend on physical environment, Societal, and Socioenvironmental factors. Physical environment include feasibility of junk food; for instance, The literature found that high consumption of snacks and junk food is higher among adolescents who have peers adopting similar eating habits [15]. Societal factors include mass media and marketing [16, 17]. Food marketing via commercial television or special channel marketing such as magazine or email text message may influence the selected food by adolescents and their eating behaviors [18]. Socioenvironmental factors include family and peer influence [19]. Family shares their behaviors and attitude with each other overtime (park, Burie 2008).

Development of DEB among adolescents has a tendency to continue during adulthood [2]. DEBs are associated with functional consequences including an increase in medical morbidity and mortality, health care utilization, and social role adjustment problems [21].

Moreover, engaging in peer groups is a prominent occurrence where adolescents try to belong to peer entities as part of exploring their social identity [22]. Erikson explained this phenomenon as exploring their individuality to avoid identity diffusion and confusion [23]. The bond within peer groups can have a positive or negative effect on adolescents' health [24]; however, DEB tends to increase within the peer group as a result of negative peer pressure [25, 26]. Adolescence is a stage of vulnerability, especially among girls, which could increase the risk of engaging in DEB because adolescents begin focusing on body image as a result of the changes related to puberty, including sudden weight gain [27]. Research on the sociocultural context of adolescents has mostly focused on media and parental pressure on adolescents, while peer pressure has received less attention [28].

Oliver and Thelen [29] made an approximation to children's perceptions of peer influence through the development of the Inventory of Peer Influence on Eating Concerns (I-PIEC) and thus suggested three domains of perception of peer pressure on DEB among boys and girls. The first domain is message pressure such as weight-related teasing (WRT) from peers about body weight [29]. The WRT has been seen as a form of bullying and peer victimization [30]. Additionally, WRT by peers has been identified as a strong predictor factor for DEB, particularly, in early adolescence [31]. Interaction between peers is the second domain, during which they share concerns about eating behaviors and body shape. Likeability pressure comprises the third domain; this is the perception of being more liked when having a thin body. One study identified message and likeability as predictors for DEB but interaction was not [32].

Although several studies excluded boys [19], messages were found to be associated with DEB among boys as well [32]. Although likability is the strongest motivation factor for DEB, messages, particularly weight-related teasing (WRT), is the most studied form of peer influence on eating behaviors and body image [33]. Of the three domains, interaction and likeability among peers are seen to be indirect factors for body satisfaction and dissatisfaction while message is found to directly influence DEB.

Previous studies found that girls who reported engaging in interaction with their friends about body weight and appearance and those who perceived likability if they were thinner among peers were more likely to develop the internalization of being thin to become beautiful, perceiving thin as the standard for beauty [33, 34]. Subsequently, this internalization of the thin ideal will influence the tendency to compare one's own body and appearance to that of others. Consequently, the internalization of thin ideal as well as body and appearance comparisons could influence the degree of body satisfaction and lead to developing DEB [19, 34].

## 2. Theoretical Framework

Two constructs of social cognitive theory [35], reinforcement and modeling, are applied in the current study to explain the pathway of influence for peer pressure on social reinforcement of DEB. Reinforcement is an important construct especially when the messages such as WRT from others, especially peers, tend to support that thin is an ideal of beauty, so this reinforces internalization of the thin ideal in the community. Moreover, teasing tends to enhance body dissatisfaction and encourage engaging in disordered eating behaviors to achieve thin and beauty of the body weight and body appearance.

According to one study, modeling is defined as “learning that occurs by virtue of witnessing another person perform a behavior” [36]. The interaction could support modeling when the adolescent talks about behavior used to achieve the thin ideal, and then they use the same unhealthy behaviors they see others perform. This may lead to the development of DEB. Moreover, the perception of being thin will increase adolescent's likeability among peers, so this can enhance DEB to achieve the thin ideal.

Jordan is an example of a developing country with low economic status that is moving towards a Western lifestyle [8]. This movement could affect the culture and norms, especially those related to eating behavior developments such as the spread and wide accessibility of fast-food restaurants, thus exposing youth to eat unhealthy food more often with their peers [37]. In spite of the increasing prevalence of DEB among Jordanian adolescents [8, 37], there is no study identifying the relationship between peer pressure and eating behaviors among adolescents from both genders in Jordan. The main aim of the current study therefore was to assess the relationship between risk for eating disorders and peer pressure among adolescent boys and girls.

## 3. Methods

### 3.1. Design

A descriptive, correlational, cross-sectional design was used to assess the relationship between peer pressures and disordered eating behaviors among Jordan's adolescents. This research and all study procedures were approved by the Institutional Review Board (IRB) at Jordan University of Science and Technology and the Ministry of Education in Jordan.

### 3.2. Population and Sample

A multistage cluster random sampling technique was used in this study to recruit junior high students aged 13 years to 16 years old from both genders from grades 8 to 10. During the first stage, a sample of four districts was selected from a list of all eight districts in northern Jordan using a simple random sampling technique.

In total, 123 Jordanian secondary schools were identified and were stratified by the type of school (16 private and 107 public). Then, the list of the public schools was stratified by gender (52 female schools and 55 male schools) with final random selection, using simple random sampling technique; of eight public schools (4 boy schools and 4 girl schools). In regards to the private schools, a total of three were selected by convenience sampling technique as the educational system in private schools in Jordan is stricter in terms of involving students in research that could affect teaching process, although it usually does not. Nonetheless, private schools were a better setting for us to conduct the online survey at as the schools are well equipped with computers and good internet access. Overall, there are no differences between schools in Jordan except for the type of school (private vs public) where the majority are academic schools. The main difference between private and public schools is the quality of services provided like transportation, scrub clothes, uniforms, and sanitations. Of the 800 eligible adolescents invited to participate in the study, 738 (92.3%) completed the online survey and about 44.7% of boys (*n*=330) and 55.3% of girls (*n*=408) were randomly selected from 8th to 10th grades from schools (*n*=11) in the northern part of Jordan, using the simple random selection technique.

## 4. Measures

### 4.1. Disordered Eating Behaviors

Eating Attitude Tests (EAT-26) questionnaire was developed to measure students' eating behavior and the associated risk/symptoms for eating disorders [38]. The EAT-26 scale assesses a broad range of DEBs such as dieting behaviors, drive for thinness, the bulimia nervosa tapped binge eating, self-induced vomiting, oral control eating, and excessive exercise [37]. The EAT-26 contains 26 items referring to various DEBs (e.g., avoid eating when I am hungry.). Each item uses six points Likert-type ranging from “always” to “never”. A score of three points was given for “always”, two points for “usually”, and one for “often”. Scores “sometimes,” “rarely,” and “never” were scored zero. This is accepted for items up to 25 items, while scoring of the item 26 is vice versa; a score of three points was given for “often,” two points for “usually,” and one point for “always”. The possible range of scores is 0 to 78 points. The participant is considered at high risk of DEB and needs further investigation when the total score is 20 points or above [38].

The EAT-26 scale has been widely used with acceptable reliability and validity in nonclinical samples of boys and girls [38]. Also, the EAT-26 was validated in the Arabic language (Cronbach's alpha value 0.80) [37] and used among Jordanian girls aged between 10 and 16 years. In the present sample, Cronbach's alpha for EAT-26 scale was 0.69 which reflects adequate internal consistency reliability.

### 4.2. Peer Pressure

The final product of the Inventory of Peer Influence on Eating Concerns (I-PIEC) Arabic version contains 16 items for males and 18 items for females [29]. Boys and girls were asked to respond to the items on a five likert-type scale from 1 to 5 (1 = never, 2 = almost never, 3 = not very often, 4 = sometimes, and 5 = a lot). The I-PIEC component from the three constructs includes message subscale which has 8 items for both genders (e.g., friends tease me or make fun of me about the size or shape of my body), interaction subscale that measured the frequency that adolescents interacted (talked, exercised, and compared bodies) about eating or body image and has 3 items for boys (e.g., boys and I compare the size and shape of our bodies) and 5 items for girls (e.g., I talk with girls about what types of food make people fat), and finally, likability subscale, which measured the degree to which adolescents believed that being thin would increase their likeability among peers. The likeability subscale for boys has 5 items (e.g., I think that boys think I would look better thinner) and 5 items for girls (e.g., If I were thinner, I think that girls would want to sit next to me more often).

The I-PIEC instrument was translated into Arabic language by an expert bilingual translator and translated back into English language by a native speaker to guarantee accurate translation. Content validity was obtained by asking two independent faculty professors in the nursing faculty, one doctor from the collage of Nutrition and one doctor from Arabic literature to validate it. The experts recommended that the original messages subscale, which has 12 items, should have 4 items deleted as they were not suitable in Arabic culture. The 4 deleted items were meant to be asked to both genders if they are at the same school. The culture in Jordan is somehow conservative and the majority of schools, especially public schools, after the age 10 years are unisex, thus boys and girls go to separate schools.

Additionally, the internal consistency reliability (Cronbach's alpha) of the instrument in the current study for girls and boys were 0.86 and 0.90, respectively. Interestingly, the I-PIEC scale was constructed for Western culture, in which the majority of schools are coeducational but Arab schools are mostly unisex. There are no reports published for the psychometric properties of the I-PIEC in Arab countries. The final I-PIEC translated Arabic version contains 16 items for boys and 18 items for girls. Boys were asked 16 items on five-point likert scale from 1 = never, 2 = almost never, 3 = not very often, 4 = sometimes, and 5 = a lot. The possible range of scores is 16–80 points. Girls were asked 18 items on five-point likert scale with possible scores ranging from 18 to 90 points.

## 5. Procedure

All students recruited for participating in the study were invited to voluntarily assent and obtain signed consent forms from their parents. On the following day, written consent forms, which were signed by parents, were collected from students by the researchers. On the day of data collection, participants were included in the study only if they agreed to participate without having any pressure from parents, teachers, or the investigator. To assure confidentiality, school teachers were not allowed to see students' answers. Before proceeding, the participants were informed that they have the right to refuse to answer any question by selecting the choice “no answer” and to withdraw from the study at any time without any penalties.

Data collection consisted of direct measurement and an online survey which was developed by the researcher using Google drive forms. Selected answers were connected to the researcher's drive directly as an excel sheet. Students who only had the desire to participate in the study were asked to complete the online Google survey during the sport and/or the art classes at the computer lab in the school.

## 6. Ethical Considerations

The research obtained approval from the Institutional Review Board (IRB) at Jordan University of Science and Technology, Jordan. All students who were recruited in the study had been asked voluntarily to sign child informed assent as well as a parental informed consent after explaining the purpose, potential risk, and procedure. They also were informed that they have the right to refuse answering any questions and withdrawing from the study at any time without any penalties. The confidentiality and anonymity were carefully protected and ensured during all stages of the study.

## 7. Data Analysis

Data were analyzed using IBM, SPSS statistics version 21. Descriptive statistics including the number and percentage were presented along with mean (standard deviation) as appropriate. Chi-square test was used to test the association between variables, and *t*-tests compared means of peer pressure values between boys and girls. Multiple logistic regression analysis was used to measure the adjusted effect of the independent variables (interaction, likability, and message) on outcomes variable, using adjusted odds ratio and 95% confidence interval with an alpha level of 0.05.

## 8. Results

Students' age ranged from 14 to 16 years (mean age (SD) = 15.06 (0.8) years). One hundred thirty-seven students were recruited from private schools (18.4%), and 601 participants were from public schools (81.6%) (Table 1).

The mean difference in peer pressure (PP), measured using the I-PIEC, between boys and girls was found to be statistically significant (*p*=0.012) (Table 2). Boys had a mean score of 1.7 (SD = 0.7) whereas girls had a mean score of 1.8 (SD = 0.7).

There were no statistically significant differences according to gender in the PP message subscale (*p*=0.626). The mean score of PP message subscale was not different among boys (*M* = 1.5, SD = 0.7) and girls (*M* = 1.5, SD = 0.8). The PP interaction subscale mean was statistically higher for girls (*M* = 2.5, SD = 1.1) versus boys (*M* = 1.8, SD = 0.9) (*p* < 0.001). The PP likeability mean scores were statistically higher for boys (*M* = 1.8, SD = 1.0) than the PP likeability subscale mean for girls (*M* = 1.5, SD = 0.9) (*p* < 0.001) (Table 3).

Among all participants, the prevalence of DEB was 23.6 % (Table 4). The percentage of DEB among girls (29.4%) was statistically higher than that among boys (16.4%) (*p* < 0.000).

Independent sample *t*-tests were used to determine if there was a difference in the means of PP between adolescents by eating behaviors (Table 5). The difference in the overall I-PIEC mean between adolescents with DEB (*M* = 2.2, SD = 0.9) and normal eating behavior states (*M* = 1.6, SD = 0.6) was statistically significant (*p* < 0.001). Within the I-PIEC subscales, message, interaction, and likeability were statistically significant. The mean score of the PP message subscale was significantly higher among adolescents with DEB (*M* = 1.9, SD = 0.9) than adolescents with normal eating behaviors (*M* = 1.4, SD = 0.6), (*p* < 0.001). Also, PP interaction mean score for adolescents with DEB (*M* = 2.7, SD = 1.2) was significantly higher than the mean score of PP interaction mean for adolescents with normal eating behaviors (*M* = 2.0, SD = 0.9), (*p* < 0.001). Furthermore, the mean score for PP likeability was significantly higher in students with DEB (*M* = 2.1, SD = 1.2) than that in students with normal eating behavior (*M* = 1.5, SD = 0.8), (*p* < 0.001).

In order to examine the factors predicting DEB, binary logistic regression analysis was conducted on the total sample (*n*=738) (Table 6). In the logistic regression model, DEB served as the dependent variables. The independent variables entered into the model as potential predictors were the three I-PIEC subscales (interaction, likeability, and message). For each unit increase in interaction scale, the risk of developing DEB also increases by 1.31 (95% CI = 1.09–1.57). For the likeability subscale, for each one-unit increase in likeability scale, the risk for developing DEB increases by 1.30 (95% CI = 1.07–1.58). However, the message PP subscale did not predict DEB (OR = 1.04, 95% CI = 0.71–1.52).

## 9. Discussion

The findings of this study reported a significant relationship between peer influence and DEB, which is consistent with previous studies [26, 32, 39, 40]. Specifically, there is a significant effect of message dimension of peer pressure on DEB; adolescents who are teased about their body weight and received negative comments on appearance have low DEB. It is interesting to note that the current study found no significant gender-related differences between response and teasing, and this is in line with a previous study [32].

The findings also indicate that interaction and likability dimensions of peer pressure are significantly associated with DEB as found also in previous studies [29, 32]. The current study also found that, while girls respond more to body comparison of their body weight and body image (interaction), boys are concerned that if they are thinner their peers will like them better (likeability). This is inconsistent with Meyer and Gast [32] finding, which found that boys respond significantly more to interaction than girls do. More research is needed to identify the differences in motivation by peers according to gender.

The current findings also showed that the interaction construct of peer pressure was the most statistically significant predictor of DEB followed by likeability construct of peer pressure; however, message did not predict DEB.

Overall, peer pressure is well documented in the literature as a significant predictor for unhealthy behaviors such as cigarette smoking and alcohol use [27, 41, 42]. The process of peer pressure on unhealthy behaviors is explained by Bandura's (1986) social cognitive theory (SCT) [32, 35]. Our study confirms the importance of SCT in exploring the influence of peer pressure on DEB. Based on SCT, peer modeling (interaction and likability) is the strongest predictor of peer pressure for DEB while peer reinforcement (message) is not.

According to the theoretical framework that we adopted in our study, a possible explanation for the strong association between peer modeling and DEB is that adolescents who reported engaging in body comparison and conversation about their appearance and body weight with their peers as well as ideas and thoughts entrenched in the mind of adolescents that they would be more accepted and popular if they were thinner and were more likely to trigger internalization of the thin ideal of beauty in the community. In turn, this indirectly could have influenced body dissatisfaction and force adolescents to mimic unhealthy eating behaviors performed by their peer [33, 43].

On the other hand, message dimension of peer pressure was not considered a predictor of DEB because it directly affects body dissatisfaction [33, 37, 44].

The findings of the current study have this implication for the primary prevention of DEB. School nurses are in the best position to conduct school-based primary prevention for DEB, particularly those who use the peer-led approach. Such nurses need to be aware that DEB is common within both genders. It is important for school nurses to educate students, their parents, and teachers about the importance of healthy eating behaviors and the considerable influence of peer context on eating behaviors. In particular, school nurses can train a group of senior students to train their peers about unhealthy eating behaviors. Moreover, school nurses are in the best position to make referrals in such cases.

A direct prevention program is suggested to focus on negative peer pressure and the outcomes of such perceived pressure. Peer-led peer prevention programs could be promising for adolescents to show how to utilize peer pressure in a positive way and help in increasing their knowledge as well as effectively changing attitude and behaviors. Gender differences need to be taken into consideration when designing prevention programs, given the differences between boys and girls in this regard.

As indirect or modeling (interaction and likeability) constructs of peer pressure seem to be the most important predictor factor for DEB, it is important to address these constructs in a school-based program. Body comparison and standards of beauty should be discussed in detail with adolescents.

Although a large and representative sample was included in this study, a cross-sectional design did not allow us to assess peer pressure in the true sense of the word. So, in future research, it is suggested to use longitudinal methodologies. For example, adolescents could be followed in three points at the beginning, middle, and the end of school years or adolescents could be followed from early to late adolescence, and more social effects such as parents and media pressure could be considered and measured.

## 10. Strengths and Limitations

Strengths and limitations should be addressed when interpreting the findings of this study. The strengths of this study include the random selection and large number of the samples which made the sample more representative. Including both boys and girls makes this study different than previous studies that focused on girls only. In addition, the survey was conducted using online survey, which eliminated the possibility of having missing data, as in the online survey, students were not able to answer the next question unless they provided a response to the current question. In addition, the error result from data entry avoided as the selected answers directly connected to the excel sheet.

Limitations of this study include the self-reporting bias. Although self-report questionnaire is the most common method of data collection, it could be affected by the subjectivity of participants. Also, the data in the current study are derived from a cross-sectional design, which cannot draw causality. The I-PIEC scale was constructed for Western culture, where the majority of schools there are coeducational, having both boys and girls in the same class. However, Arab schools are mostly unisex, thus the division of schools based on gender is another limitation for studying how adolescents feel with their other sex peers. Finally, the design of this research was descriptive cross-sectional in which the association between variables is unclear, and thus causality cannot be established.

## 11. Conclusions

In summary, the current study is the first study highlighting the important effect of peer pressure context on unhealthy eating behaviors and risk of eating disorders in Jordan. Most of the research studied message form of peer pressure; however, the present study indicates the most significant predictor is interaction and likeability for DEB. Findings have implications for the primary prevention of eating behavior disorders. Finally, future research should assess the impact of students' disordered eating behaviors on peers' health and academic performance [45].

## Figures and Tables

**Table 1:** Demographic characteristics of the students (*N*=738).

Variables	Total, *N* (%)	Boys, *n* (%) 330 (44.7)	Girls, *n* (%) 408 (55.3)
Age in year (mean (SD) = 15.06 (0.8))			
** **14	218 (29.5)	76 (23.0)	142 (34.8)
** **15	263 (35.6)	138 (41.8)	125 (30.6)
** **16	257 (34.8)	116 (35.2)	141 (34.6)

School			
** **Public	601 (81.4)	270 (81.8)	331 (81.1)
** **Private	137 (18.6)	60 (18.2)	77 (18.9)

**Table 2:** Association of gender with perception of peer pressure (*N*=738).

	Gender		*t*-value	*P* value
	Boys (mean (SD))	Girls (mean (SD))		
Peer pressure	1.7 (0.7)	1.8 (0.7)	**−2.52**	**0.012**
Message	1.5 (0.7)	1.5 (0.8)	**−0.48**	**0.626**
Interaction	1.8 (0.9)	2.5 (1.1)	**−8.34**	**<0.001**
Likability	1.8 (1.0)	1.5 (0.9)	**3.46**	**<0.001**

**Table 3:** Correlation of gender with perception of peer pressure (*N*=738).

	Gender		*P* value
	Boys	Girls	
	Mean (SD)	Mean (SD)	
Perception peer pressure	1.7 (0.7)	1.8 (0.7)	0.012
Message	1.5 (0.7)	1.5 (0.8)	0.626
Interaction	1.8 (0.9)	2.5 (1.1)	0.0001
Likability	1.8 (1.0)	1.5 (0.9)	0.001

**Table 4:** Distribution of study participants by eating behaviors (EB) status and gender (*N*=738).

Eating behavior status			*P* value
	EAT: 26 < 20, *N*(%)	EAT: 26 ≥ 20, *N*(%)	
Gender			
Boys	276 (83.6)	54 (16.4)	0.0001
Girls	288 (70.6)	120 (29.4)	

**Table 5:** Association of eating behaviors with the perception of peer pressure in school students in northern Jordan (*N*=738).

	Eating behavior (mean (SD))		*t*-value	*P* value
	Normal	Disordered		
Peer pressure	1.6 (0.6)	2.2 (0.9)	−9.56	0.0001
Message	1.4 (0.6)	1.9 (0.9)	−7.86	0.0001
Interaction	2.0 (0.9)	2.7 (1.2)	−7.30	0.0001
Likeability	1.5 (0.8)	2.1 (1.2)	−7.37	0.0001

**Table 6:** Logistic regression analysis of factors associated with disordered eating attitudes and behaviors independent (*n*=738).

Independent variable	Odds ratio	95% C.I.	*P* value
*Gender*			
Boys			
Girls	1.54	0.94–2.52	**0.080**

*I-PIEC*			
Interaction	1.31	1.09–1.57	**0.003**
Likeability	1.30	1.07–1.58	**0.007**
Message	1.04	0.71–1.52	**0.816**

## Data Availability

The data is secured with the first author and is available for anyone to have a look at.
